# Relationship between the severity of endometriosis symptoms (dyspareunia, dysmenorrhea and chronic pelvic pain) and the spread of the disease on ultrasound

**DOI:** 10.1186/s13104-020-05388-5

**Published:** 2020-11-25

**Authors:** Elham Kor, Seyed Reza Saadat Mostafavi, Zahra Ahmadian Mazhin, Adeleh Dadkhah, Anis Kor, Shirin Habibi Arvanagh, Shima Ghafourian Noroozi, Ghazal Sadri

**Affiliations:** Department of Radiology, Hazrat Rasoul Akram Hospital, Iran University of Medical Sciences, Tehran, Iran

**Keywords:** Endometriosis, DIE, Transvaginal ultrasonography

## Abstract

**Objectives:**

About 10–15% of women of childbearing age have endometriosis. The present study aimed to investigate the relationship between the severity of symptoms of endometriosis and the spread as well as the stage of the disease on ultrasonography. The present cross-sectional study evaluates the relationship between the severity of endometriosis symptoms and the spread of disease on ultrasonography in patients with endometriosis.

**Results:**

Considering different analyses, the cumulative size of posterior deep infiltrative endometriosis (DIE) (less than 1 cm) is significantly correlated with minimal severity of dyspareunia and chronic pelvic pain. The incidence of dyspareunia was more prevalent in patients with complete stenosis of Douglas pouch than those with incomplete stenosis. Furthermore, the incidence of severe and very severe pain in patients with Douglas pouch stenosis is relatively higher than that in patients without stenosis. Only dyspareunia is related to the stage of endometriosis, and patients with dyspareunia are five times more at risk of a higher stage of the disease. The severity of dyspareunia is related to the stage of endometriosis and the severity of Douglas pouch stenosis. The results showed a correlation between chronic pelvic pain and r-ASRM score (revised American Society for Reproductive Medicine score).

## Introduction

Endometriosis refers to the extrauterine presence of endometrial glands and stroma leading to reactive inflammation and fibrosis [1].

Endometriosis affects 10–15% of women of childbearing age. Most of the patients are in the 18–25 age range [2]. The prevalence of the disease is even higher in symptomatic individuals (infertile individuals = 50%, patients with chronic pelvic pain = 50–90%) [3, 4]. Endometriosis may cause severe painful symptoms such as dysmenorrhea, dyspareunia, and chronic pelvic pain while doing daily activities. The painful and chronic symptoms of this disease result in a poor quality of life in most of the patients [5–7]. The ovaries are one of the most common sites of endometriosis. Ovarian endometrioma is a pathognomonic manifestation of endometriosis. Endometrioma is a type of cyst formed from bloody ectopic endometrial glands inside the ovary. This cyst has fibrous capsules and contains blood products [1].

**Fig. 1:**
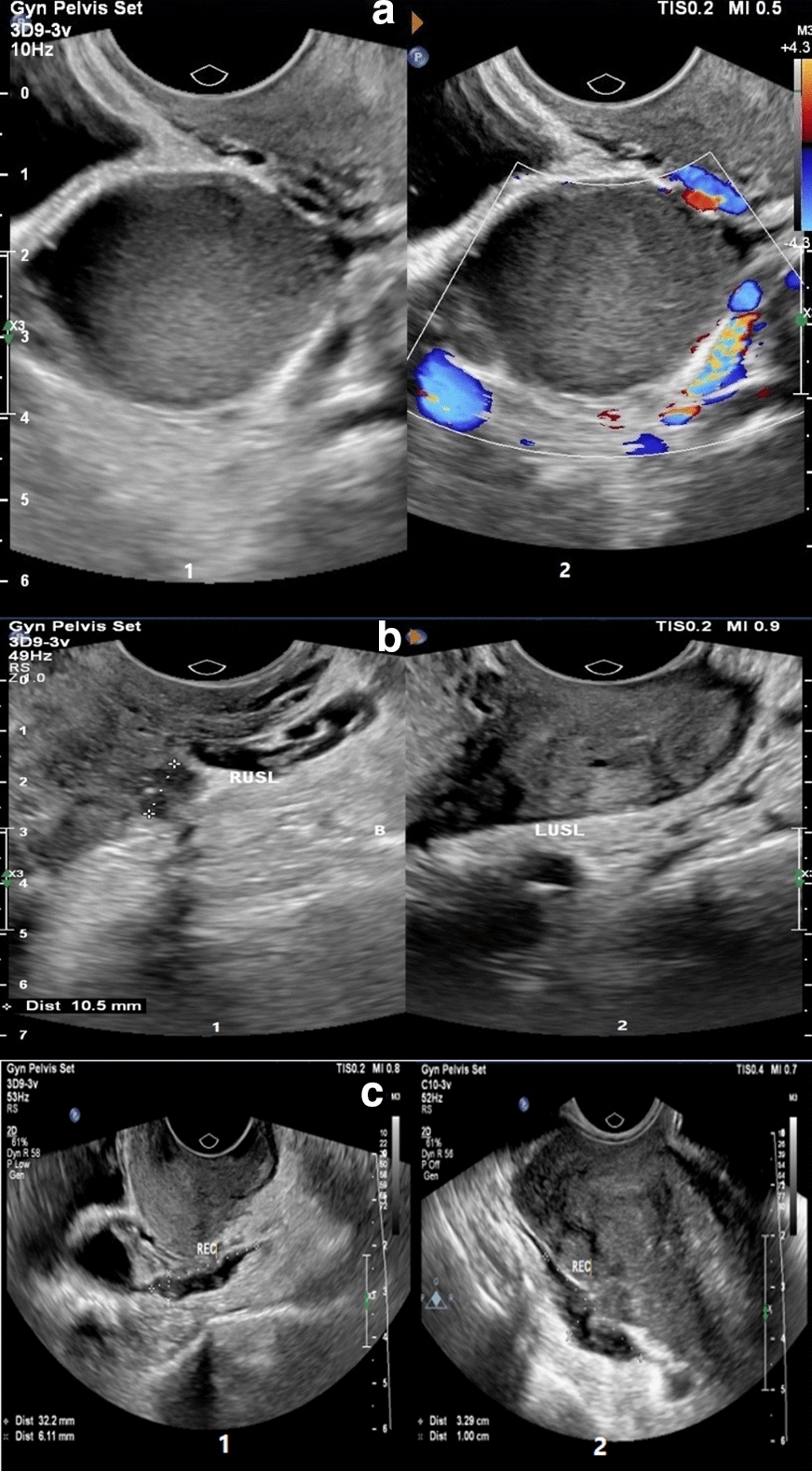
**a** Typical ovarian endometrioma in a woman with long-standing chronic pelvic pain and dysmenorrhea (1, 2) gray-scale (1) and color Doppler (1) TVS images of right ovary demonstrate a unilocular cyst containing homogeneous low-level echoes and no internal vascularity at color Doppler US (classic appearance of an ovarian endometrioma). **b** USL DIE in a woman with severe pelvic pain and dyspareunia for 10 years with a history of stage IV endometriosis who was confirmed to have extensive endometriosis at laparoscopy. (1) sagittal gray-scale TVS image shows irregular thickening of the right USL associated 10 mm endometriosis nodule in proximal. (2) Also, a moderate thickening of left USL has been shown. **c** Bowel DIE in two women. (1) Sagittal gray-scale TVS image in a woman with severe dysmenorrhea shows a hypoechoic nodule involving the serosal layer in the lower rectum. (2) Transverse gray-scale TVS images in a woman with chronic pelvic pain and cramping, show a hypoechoic nodule in the rectosigmoid junction with severe adhesion to the posterior of the uterus fundus

Deep infiltrative endometriosis (DIE) is a specific form of endometriosis which refers to endometrial implants above 5 mm penetrating the peritoneal surface. These implants are highly active and almost associated with pelvic pain symptoms. The most common effected areas for these implants include posterior areas, uterosacral ligament (USL), torus uterus (the retro-cervical part of uterine where uterosacral ligaments join there), posterior wall of the vagina, and posterior wall of the rectum [8]. Although clinical findings may suggest the disease, imaging is needed for definitive diagnosis [4]. According to The American College of Obstetricians and Gynecologists (ACOG), transvaginal ultrasonography (TVS) is the first method found for studying endometriosis, and magnetic resonance imaging (MRI) is used if rectovaginal or bladder involvement is suspected [1, 4]. The value of ultrasound in the diagnosis of endometriosis has been confirmed. Several papers have shown that TVS is comparable and even better than MRI [9]. TVS is highly specific for the detection of DIE in uterosacral ligaments, rectovaginal septum, vagina, and bladder [10]. The classic appearance of endometrioma in ultrasound examination is homogenous, and hypoechoic cystic ovarian lesion with low-level internal echo and without internal blood flow is displayed [1, 11] (Fig. 1a).

Accurate mapping of disease spread is critical for determining treatment strategy; however, the accuracy of TVS is confirmed. The dynamism of ultrasound increases its value and evaluates areas not examined by other imaging modalities.

Considering the importance of endometriosis and its relatively high prevalence among women as well as the wide range of endometriosis symptoms (including asymptomatic to severe life-threatening pain), researchers of this study sought to study the relationship between the various symptoms of the disease and the spread of its anatomical involvements. Moreover, accurate diagnosis of disease spread using non-invasive methods can be effective in the treatment and follow-up of patients. TVS is the most accessible imaging method as well as the selective modality for differentiating endometrioma from other cystic lesions. The present study seeks to identify cases of endometriosis which do not require invasive treatment or laparoscopic diagnosis. Furthermore, preoperative non-invasive mapping and grading of disease in patients requiring surgery can help improve surgical outcomes.

## Main text

### Methods

This cross-sectional study evaluates the relationship between the severity of endometriosis symptoms (dyspareunia, dysmenorrhea, and chronic pelvic pain) and the spread of disease on ultrasonography in patients with endometriosis. Target population included women with endometriosis symptoms (dyspareunia, dysmenorrhea, and chronic pelvic pain), referring to the radiology ward of Hazrat Rasool Akram Hospital in Tehran from 2018 to 2020. All patients had a complete gynecological examination by a gynecologist. None of the patients had a previous history of surgery due to endometriosis. Inclusion criterion included patients with endometriosis symptoms and a diagnosis of endometriosis based on ultrasound examination and laparoscopic confirmation. The definitive diagnosis of all patients during this period was confirmed by laparoscopy and pathological examination. The required information was extracted from the patient's ultrasound examination report and questionnaires then included in the special forms designed for this purpose. The severity of pain was determined by Point Pain Numbered Scale 11 (NRS11) [12]. Patients filled out self-administered questionnairesand ranked their pain ranging from zero to ten. Exclusion criteria included other causes of pelvic pain such as pelvic inflammatory disease (PID), pelvic varices, ovarian cysts except for endometrioma, gastrointestinal and urinary problems, and gynecological surgery history. Ultrasound examinations were done by Philips Affiniti 70 ultrasound *machine* with three models including C-10 3v, C5-1, and L12-3 examining vaginal probe (10-3 MHz), curve probe (5–1 MHz) and linear probe (12–3 MHz), respectively. All ultrasound examinations were performed by an experienced radiologist who was unaware of the type and severity of the patient’s pain. It wasdone based on the systematic protocol introduced by the International Deep Endometriosis Analysis (IDEA) Consensus Group [13]. Ultrasound examination includes the evaluation of uterine and adnexa, the assessment of the sliding sign, dedicated search for DIE, and the detection of sonographic soft markers such as localized tenderness [13]. The disease stage was scored based on the r-ASRM classification [14]. For this purpose, systematic ultrasound data with endometriosis protocol confirmed by laparoscopic data were used.

Results were statistically analyzed using the SPSS 24. To describe quantitative data, mean and standard deviation and for qualitative data, frequency and percentage were used. Accordingly, Chi-square and Fisher's statistical tests were used to investigate the association of any of the symptoms such as dysmenorrhea, dyspareunia, and pelvic pain with endometrioma, Douglas pouch stenosis, intestinal involvement, and stage of the disease.. Finally, to examine the relationship between each symptom and the stage of the disease, a logistic regression test for sequential data was used.

## Results

A total of 296 patients underwent ultrasonography based on endometriosis protocol at the Department of Radiology between January 2018 and August 2020. Out of 296 patients, 142 cases were excluded as follows: patients with incomplete ultrasound report (0.67%), with amenorrhea (n = 5, 1.6%); with the previous resection of DIE (n = 7, 2.3%), with an insufficient description of the posterior DIE (n = 10, 3.3%), and patients who do not intend to participate in the study (n = 118, 39.8%). Finally, 154 patients remained to be examined. The demographic characteristics and clinical data of patients are presented. The mean age of participants was 32.4 ± 6.2 years. The presence and severity of pain based on the disease stages (according to r-ASRM classification) are presented in Table 1. The severity of dysmenorrhea, dyspareunia, and chronic pelvic pain considering theextension of the disease are presented in Table 2. Typical ovarian endometrioma, uterosacral ligament, and bowel involvement with DIE is displayed in Fig. 1a–c, respectively.

**Table 1:** Presence and severity of pain syndrome according to the stage of endometriosis

Pain syndrome	Minimal No (%)	Mild No (%)	Moderate No (%)	Severe No (%)	p value
Dysmenorrhea					
Stage I	0	1 (8.3)	2 (16.7)	9 (75)	0.61
Stage II	0	0	3 (30)	7 (70)	
Stage III	0	9 (15.8)	18 (31.6)	30 (52.6)	
Stage IV	4 (5.5)	7 (9.6)	22 (30.1)	40 (54.8)	
Dyspareunia					
Stage I	5 (45.5)	0	4 (36.4)	2 (18.2)	0.48
Stage II	4 (44.4)	0	3 (33.3)	2 (22.2)	
Stage III	21 (47.7)	8 (18.2)	7 (15.9)	8 (18.2)	
Stage IV	20 (34.5)	12 (20.7)	17 (29.3)	9 (15.5)	
Chronic pelvic pain					
Stage I	7 (58.3)	0	2 (16.7)	3 (25)	0.05
Stage II	5 (50)	0	5 (50)	0	
Stage III	35 (60.3)	9 (15.5)	10 (17.2)	4 (6.9)	
Stage IV	32 (44.7)	22 (30.6)	13 (18.1)	5 (6.9)	

*NO *number

**Table 2:** The severity of dysmenorrhea, dyspareunia, and chronic pelvic pain according to the extension of disease

Variable	Total	Minimal No. (%)^a^	Mild No. (%)^a^	Moderate No. (%)^a^	Severe No. (%)^a^	p
Dysmenorrhea severity						0.8
The cumulative size of DIE implants						
< 1 cm	22	0	2 (9.1)	9 (40.9)	11 (50)	
1–3 cm	47	1 (2.1)	4 (8.5)	19 (40.4)	23 (48.9)	
> 3 cm	41	2 (4.9)	4 (9.8)	11 (26.8)	24 (58.5)	
The cumulative surface of superficial peritoneal implants						0.48
0	142	4 (2.8)	16 (11.3)	40 (28.2)	82 (57.7)	
< 3 cm	5	0	1 (20)	3 (60)	1 (20)	
> 3 cm	5	0	0	2 (40)	3 (60)	
Endometrioma						0.53
None	28	0	2 (7.1)	5 (17.9)	21 (75)	
Unilateral	78	3 (3.8)	12 (15.4)	22 (28.2)	41 (52.6)	
Bilateral	51	1 (2)	3 (5.9)	18 (35.3)	29 (56.9)	
Cumulative size of endometriomas						0.31
0	28	0	2 (7.1)	5 (17.9)	21 (75)	
≤ 3 cm	26	2 (7.7)	5 (19.2)	6 (23.1)	13 (50)	
> 3 cm	100	2 (2)	10 (10)	34 (34)	54 (54)	
Size of the largest endometrioma						0.69
0	26	0	2 (7.7)	5 (19.2)	19 (73.1)	
≤ 3 cm	43	2 (4.7)	5 (11.6)	14 (32.6)	22 (51.2)	
> 3 cm	85	2 (2.4)	10 (11.8)	26 (30.6)	47 (55.3)	
Douglas obliteration						0.42
Absent	21	0	3 (14.3)	7 (33.3)	11 (52.4)	
Partial	53	3 (5.7)	7 (13.2)	11 (20.8)	32 (60.4)	
Complete	75	1 (1.3)	7 (9.3)	27 (36)	40 (53.3)	
Cumulative size of posterior DIE						0.7
< 1 cm	22	0	2 (9.1)	9 (40.9)	11 (50)	
1–3 cm	46	1 (2.2)	3 (6.5)	19 (41.3)	23 (50)	
> 3 cm	40	2 (5)	4 (10)	10 (25)	24 (60)	
Sub peritoneal extension						0.82
Sub-peritoneal only	92	2 (2.2)	10 (10.9)	27 (29.3)	53 (57.6)	
Rectal	51	2 (3.9)	5 (9.8)	16 (31.4)	28 (54.9)	
Vaginal	1	0	0	1 (100)	0	
Both rectal and vaginal	2	0	0	0	2 (100)	
Dyspareunia severity						
Cumulative size of DIE implants						
< 1 cm	16	10 (62.5)	3 (18.8)	1 (6.3)	2 (12.5)	0.05
1–3 cm	36	13 (36.1)	4 (11.1)	9 (25)	10 (27.8)	
> 3 cm	37	13 (34.2)	9 (23.7)	13 (34.2)	2 (7.9)	
Cumulative surface of superficial peritoneal implants						
0	113	46 (40.7)	19 (16.8)	27 (23.9)	21 (18.6)	0.48
< 3 cm	4	1 (25)	0	3 (75)	0	
> 3 cm	5	3 (60)	1 (20)	1 (20)	0	
Endometrioma						
None	26	11 (42.3)	2 (7.7)	9 (34.6)	4 (15.4)	0.14
Unilateral	61	23 (37.7)	13 (21.3)	11 (18)	14 (23)	
Bilateral	40	18 (45)	7 (17.5)	12 (30)	3 (7.5)	
Cumulative size of endometriomas						
0	26	11 (42.3)	2 (7.7)	9 (34.6)	4 (15.4)	0.52
≤ 3 cm	22	10 (45.5)	3 (13.6)	7 (31.8)	2 (9.1)	
> 3 cm	76	29 (38.2)	16 (21.1)	16 (21.1)	15 (19.7)	
Size of the largest endometrioma						
0	24	10 (41.7)	1 (4.2)	9 (37.5)	4 (16.7)	0.30
≤ 3 cm	36	16 (44.4)	5 (13.9)	7 (19.4)	8 (22.2)	
> 3 cm	64	24 (37.5)	15 (23.4)	16 (25)	9 (14.1)	
Douglas obliteration						
Absent	16	7 (43.8)	2 (12.5)	5 (31.3)	2 (12.5)	0.06
Partial	48	12 (25)	10 (20.8)	18 (37.5)	8 (16.7)	
Complete	56	29 (51.8)	8 (14.3)	8 (14.3)	11 (19.6)	
Cumulative size of posterior DIE						
< 1 cm	16	10 (62.5)	3 (18.8)	1 (6.3)	2 (12.5)	0.04
1–3 cm	35	12 (34.3)	4 (11.4)	9 (25.7)	10 (28.6)	
> 3 cm	37	12 (32.4)	9 (24.3)	13 (35.1)	3 (8.1)	
Sub peritoneal extension						
Sub-peritoneal only	72	35 (48.6)	8 (11.1)	15 (20.8)	14 (19.4)	0.07
Rectal	42	13 (31)	11 (26.2)	13 (31)	5 (11.9)	
Vaginal	1	0	0	0	1 (100)	
Both rectal and vaginal	2	1 (50)	0	1 (50)	0	
Chronic pelvic pain severity						
The cumulative size of DIE implants						
< 1 cm	23	15 (65.2)	6 (26.1)	1 (4.3)	1 (4.3)	0.07
1–3 cm	46	24 (52.2)	6 (13)	13 (28.3)	3 (6.5)	
> 3 cm	41	19 (46.3)	14 (34.1)	5 (12.2)	3 (7.3)	
The cumulative surface of superficial peritoneal implants						
0	142	74 (52.1)	27 (19)	30 (21.1)	11 (7.7)	0.26
< 3 cm	5	2 (40)	3 (60)	0	0	
> 3 cm	5	3 (60)	1 (20)	0	1 (20)	
Endometrioma						
None	28	13 (46.6)	4 (14.3)	8 (28.6)	3 (10.7)	0.25
Unilateral	79	44 (55.7)	15 (19)	13 (16.5)	7 (8.9)	
Bilateral	50	22 (44)	16 (32)	10 (20)	2 (4)	
The cumulative size of endometriomas						
0	28	13 (46.6)	4 (14.3)	8 (28.6)	3 (10.7)	0.68
≤ 3 cm	26	15 (57.7)	4 (15.4)	5 (19.2)	2 (7.7)	
> 3 cm	100	51 (51)	25 (25)	17 (17)	7 (7)	
Size of the largest endometrioma						
0	26	13 (50)	2 (7.7)	8 (30.8)	3 (11.5)	0.38
≤ 3 cm	42	21 (50)	10 (23.8)	7 (16.7)	4 (9.5)	
> 3 cm	86	45 (52.3)	21 (24.4)	15 (17.4)	5 (5.8)	
Douglas obliteration						
Absent	21	13 (61.9)	2 (9.5)	4 (19)	2 (9.5)	0.5
Partial	52	22 (42.3)	15 (28.8)	10 (19.2)	5 (9.6)	
Complete	76	43 (56.6)	14 (18.4)	14 (18.4)	5 (6.6)	
Cumulative size of posterior DIE						
< 1 cm	23	15 (65.2)	6 (26.1)	1 (4.3)	1 (4.3)	0.03
1–3 cm	45	24 (53.3)	5 (11.1)	13 (28.9)	3 (6.7)	
> 3 cm	40	18 (45)	14 (35)	5 (12.5)	3 (7.5)	
Sub peritoneal extension						
Sub-peritoneal only	91	49 (53.3)	18 (19.6)	17 (18.5)	8 (8.7)	0.93
Rectal	51	25 (49)	12 (23.5)	11 (21.6)	3 (5.9)	
Vaginal	1	1 (100)	0	0	0	
Both rectal and vaginal	2	1 (50)	1 (50)	0	0	

^a^Represents the number and percentage of women with degree of severity of dysmenorrhea, dyspareunia, and chronic pelvic pain

Of the 154 women studied, 150 (48.7%) had dysmenorrhea, 75 (97.4%) had dyspareunia, and 75 (48.7%) had chronic pelvic pain.

The cumulative size of posterior DIE is (less than 1 cm) significantly correlated with minimal severity of dyspareunia (p = 0.04) and chronic pelvic pain (p = 0.03). Patients with complete stenosis of Douglas pouch had higher dyspareunia than those with incomplete Douglas pouch stenosis. Further, the incidence of severe and very severe pain in patients with Douglas pouch stenosis is relatively higher than that in patients without stenosis.

Using logistic regression analysis, it can be concluded that only dyspareunia is related to the stage of the disease so that patients with dyspareunia are 5 times more at risk of a higher stage of the disease.

## Discussion

Endometriosis is one of the challenges for women with pelvic pain and infertility. Chronic pelvic pain affects about 15% of women of childbearing age and reduces their quality of life [15]. In the present study, the mean age of patients was 32.4 years, and the disease was highly severe in less than half of the cases, which is consistent with results of Khawaja et al. [16]. The infertility rate in women with endometriosis in the present study was 37.3%, which is consistent with the study of Radhika et al. [17]. Bellelis et al. [18] reported infertility rates as 31.5 and 40%, respectively.

This study aimed to explore the relationship between the severity of endometriosis symptoms (dyspareunia, dysmenorrhea, and chronic pelvic pain) and the spread of the disease. The results show that only the severity of dyspareunia is related to the stage of endometriosis, and the odds ratio of endometriosis was 0.24 in the absence of dyspareunia. In previous studies, many attempts have been made to clarify the association of the type and location of lesions and the spread of the disease with the severity and symptoms of the disease, which has had no consensus on results [19].

The growth of nerve fibers in ectopic implants is considered as the mechanism of severe pain in endometriosis [20–22]. According to Varecellini et al.’s study [19], dysmenorrhea, as one of the most common symptoms of endometriosis, was associated with atypical and popular implants. Fedele et al. [23] reported the association of dysmenorrhea with advanced stages of endometriosis. Chapron et al. [24], showed that dysmenorrhea was associated with the size and depth of the lesions. However, in other studies, there was no relationship between menstrual pain and endometriosis [25], which shows that other factors cause this pain, and merely examining the appearance of implants can reveal the true nature of the disease.

In the present study, there was no relationship between dysmenorrhea and chronic pelvic pain, and disease severity. The results of this study are inconsistent with those of Varcellini et al. [19], who found a significant association of disease severity and dysmenorrhea with chronic pelvic pain. Although their estimated odds ratio for dysmenorrhea and chronic pelvic pain (1.33 and 1.01, respectively) was very close to 1 and does not indicate any strong correlation. A slight change in the sample volume possibility causes the missing of the confidence interval.

Results of the present study suggested that there is a significant relationship between dyspareunia and posterior DIE lesion, and has been strongly confirmed in other studies. Anatomically, the most stretched area during intercourse is the retro-cervical area [26], which indicates the organic nature of this pain. Anaf et al. [27] showed a histological relationship between nerves and endometriotic foci in retro-cervical nodules [28]. Besides, Douglas stenosis was significantly associated with dyspareunia in the present study, which has been reported in almost all previous studies. Varcellini et al. reported a strong association between Douglas pouch lesions and dyspareunia [19].

Studies show that the association between ovarian endometriosis and dysmenorrhea has conflicting results. Although endometrioma was common among the patients in the present study (83.4%), no significant association was found between endometrioma and dysmenorrhea, which is consistent with the results of some researchers (Radhika et al. [17], Porpora et al. [29], Chapron et al. [24], and Koninckx et al. [30]). In contrast, Muzii et al. [31] and Fedele et al. [23] showed thatthe association between endometrioma and pelvic pain was significant, which is inconsistent with the results of the present study.

## Conclusion

The results of the present study show that the severity of dyspareunia is related to the stage of endometrioma and the severity of Douglas pouch stenosis. The present study shows the correlation between chronic pelvic pain and r-ASRM score. Additional prospective studies are needed to validate the results of this study.

### Limitations

Some aspects of this study that are affected by cultural considerations or moral codes includedthe frequency and details of sexual activity and symptoms like dyspareunia, which may cause biases in data collection.

## Data Availability

All required data are mentioned in the paper. Any more data are available upon request from the corresponding author.

## Abbreviations

DIE: Deep infiltrative endometriosis
USL: Uterosacral ligament
ACOG: Obstetricians and gynecologists
TVS: Transvaginal ultrasonography
